# Solving yeast jigsaw puzzles over a glass of wine: Synthetic genome engineering pioneers new possibilities for wine yeast research

**DOI:** 10.15252/embr.201745231

**Published:** 2017-10-23

**Authors:** Isak S Pretorius

**Affiliations:** ^1^ Macquarie University Sydney NSW Australia

**Keywords:** S&S: Economics & Business, S&S: Technology, Synthetic Biology & Biotechnology

## Abstract

The Synthetic Yeast Genome Project will give scientists a tool for understanding the biological intricacies of eukaryotes and for synthetic biology. Wine researchers will greatly benefit from this project to build new wine yeast strains.

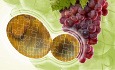

More than 7,000 years ago, when grapes and yeast joined forces for the first time to lift the spirit of humankind, the ancients imbibed with joy, unknowingly celebrating the beginnings of one of the world's oldest biotechnological processes. The first “magical” juice came from spontaneously fermented grapes cultivated in the Zagros Mountains of Ancient Persia and the Caucasus Mountain Range between the Black and Caspian Seas. The “mystical art” through which sugary, bland‐tasting grape juice is turned into flavoursome wine with hedonic and preservative properties, quickly spilled over into neighbouring regions of Mesopotamia, Anatolia, Egypt, Phoenicia, Greece and the Mediterranean Basin 1.

As European seafarers set sail to discover and explore far‐off continents, their ships carried both the “fermenting knowledge” of winemaking and the “geminating seeds” of yeast biotechnology.

Following colonisation by the Greeks, Phoenicians and Romans, winemaking spread throughout Europe and became embedded in the diet and cultural activities of both the aristocracy and proletariat. Roman potters developed large earthenware pots for storage and transport. The Gauls taught the Romans how to fashion barrels from wood, and oak barrels became the vessel of choice for yeast cells to ferment grape must into wine—a skill that survived the Roman Empire and the Dark Ages of economic and cultural decay.

With the dawn of the Age of Enlightenment, geographic exploration was accompanied by a search for scientific knowledge. As European seafarers set sail to discover and explore far‐off continents, their ships carried both the “fermenting knowledge” of winemaking and the “geminating seeds” of yeast biotechnology. A century before the French biochemist, Louis Pasteur, zoomed in to the bubbling and frothing content of oak barrels and discovered that tiny yeast cells were responsible for the fermentation of grape juice into wine, the Berkshire cartographer, John Spilsbury, zoomed out to reveal the “Big Picture” of the British Empire in 1767 through the invention of a “dissected map”. Spilsbury pasted maps onto wood, cut them into small pieces and reconstituted a jigsaw puzzle of the world. Ever since, the jigsaw puzzle evolved into a problem‐solving recreational pastime and educational toy.

## Solving scientific puzzles

Oddly, Pasteur and Spilsbury have much in common. In their indomitable quest to solve challenging problems, both were interested in uncovering every bit of detail of the “problem” and in piecing the “Big Picture” together. Their approach of seeking to understand the “fundamentals” in the context of potential application served us well through many centuries of scientific endeavour and remains the most powerful dynamo of technological and societal progress today. This statement also applies to the emerging science of synthetic biology.

**Figure 1:**
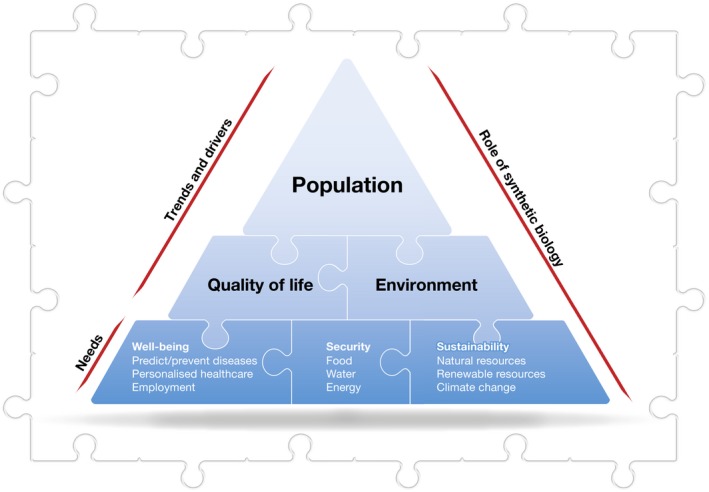
**The needs and trends associated with the growing global population require solutions to a wide variety of grand challenges linked to human well‐being, security and the sustainability of the environment**.

There is no doubt that synthetic biology technologies will be crucial to solve the puzzling challenges of a world with dwindling finite resources and a rapidly growing and ageing global population. Maximising the bioeconomy—that is, the economic activity derived from scientific advances and innovations in biotechnology and, in particular, the engineering of biology and biomanufacturing—will be one key strategy. However, assessing current bioeconomical trends and finding solutions for the grand challenges are like trying to solve a complex jigsaw puzzle without all the pieces in the box (Fig 1). The best approach is to frame human futures—improved quality of life—in a planetary context: a sustainable environment. Put differently, start by separating the puzzle's edges—well‐being, security and sustainability—from the middle pieces, such as health, food, water, energy, employment and economy.

There is no doubt that synthetic biology technologies will be crucial to solve the puzzling challenges of a world with dwindling finite resources and a rapidly growing and ageing global population.

**Figure 2:**
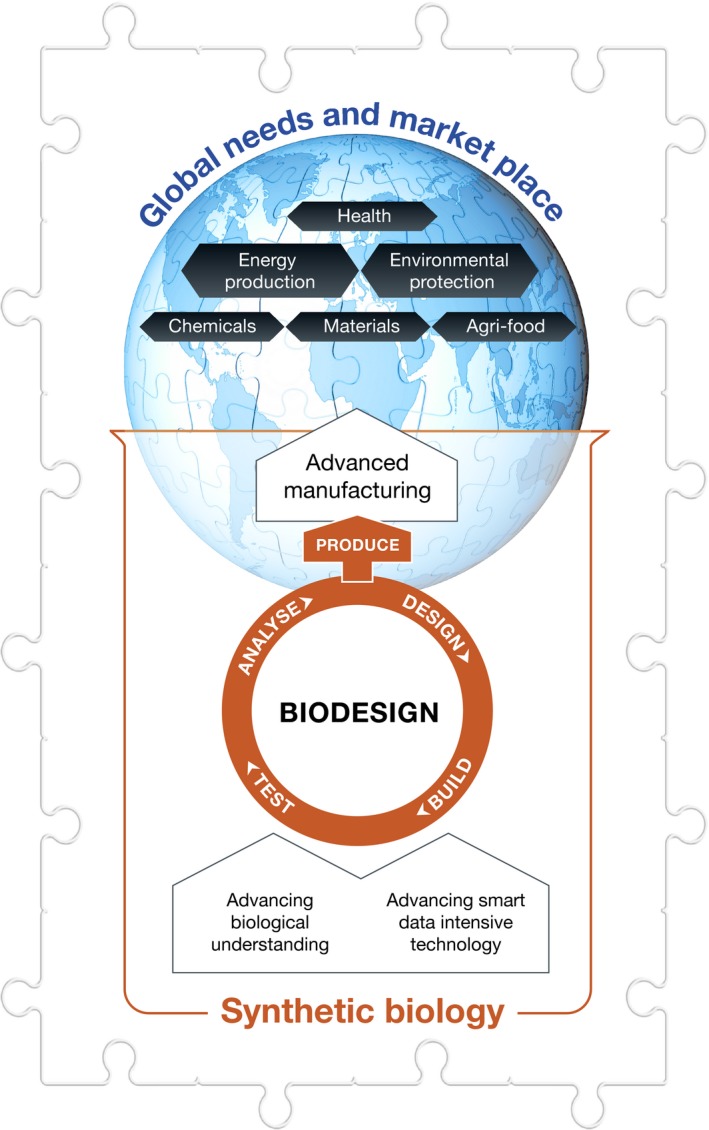
**Biodesign and biomanufacturing underpinned by synthetic biology technologies are key success factors in the new bioeconomy**.

Once the frame of the puzzle is pieced together, the middle pieces can be sorted by colour and a more complete picture emerges. These pieces include: prediction and prevention of diseases; affordable healthcare; adequate access to clean water and safe, nutritious foods; energy‐rich molecules for renewable biofuels and novel bioenergy resources; bioremediation of polluted environments and improved land use; biodegradable pesticides and sustainable, environmentally friendly industrial chemicals; and continuous workforce training in biodesign and biomanufacturing for the new bioeconomy 2. Bio‐based designing, bioengineering and advanced biomanufacturing to meet global needs depend on the development of our biological understanding and smart data‐intensive technologies (Fig 2).

The emerging discipline of biodesign—synthetic biology—builds on the rich legacies of several branches of biology—including genetics, molecular biology and systems biology—biomolecular platforms, chemical and physical sciences, mathematical and computational sciences, data science and bioinformatics, as well as engineering and information technology 2. The capability to engineer biology resulted in the development of high‐throughput analytical technologies and rapid DNA sequencing, synthesis and editing technologies fast‐tracked by automated platforms in genome foundries. These advances are making precision genome engineering faster, cheaper and more accurate by the day.

**Figure 3:**
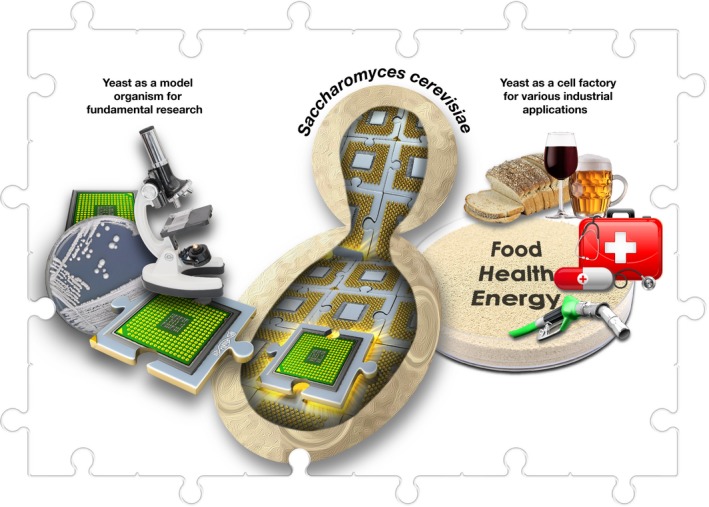
**The multi‐purpose yeast,** ***Saccharomyces cerevisiae*** **, is the best‐studied eukaryotic model organism and the most used microbe in the fermentation industry**.

In this context, the well‐studied, food‐grade yeast, *Saccharomyces cerevisiae*, has become a legacy eukaryotic “chassis” for synthetic biology (Fig 3). In synthetic biology, the engineering term *chassis* refers to the organism that serves as a framework to physically accommodate new biological parts (genes), devices (gene networks) and modules (biosynthetic pathways) to (re)design biological systems (cells and organisms) 2. *Saccharomyces cerevisiae* has a long history as a model organism for fundamental academic research as well as being a workhorse for a wide range of industrial applications. Based on this track record, it is now the preferred “cell factory” of semi‐synthetic products, such as artemisinic acid, a precursor of the potent anti‐malarial artemisinin, as well as food ingredients, including vanillin, resveratrol, saffron, stevia and nootkatone 2. The successful use of *S. cerevisiae* to produce these commercial products has moved synthetic biology from the “laboratory” to the “field”, thereby changing the term “genetically modified organism” (GMO) to “semi‐synthetic organism” (SSO) 1.

## Building the ultimate Yeast 2.0

Recent commercial advances with semi‐synthetic yeast cell factories have tossed a fresh pile of jigsaw pieces of a highly complex puzzle on the discussion tables of scientists, industry practitioners, policymakers, regulators, governments, consumers and society at large. One approach to help solve this puzzle is to create a fully man‐made genome for *S. cerevisiae* so that we can better understand the biological intricacies of eukaryotic SSOs and be able to more accurately predict and control the practical outcomes of *genome engineering—*as opposed to individual gene‐based *genetic engineering*. This is the primary purpose of the international Synthetic Yeast Genome Project, known as Yeast 2.0 or Sc2.0. This ambitious collaborative project is guided by an agreed and legally binding policy statement on key issues, such as social benefits, intellectual property, safety and governance 3, 4.

**Figure 4:**
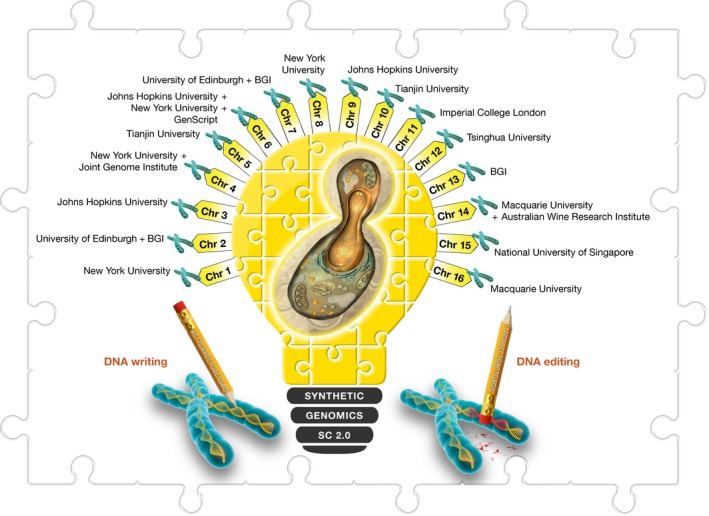
**A dozen research laboratories in five countries—** **USA** **,** **UK** **, China, Singapore and Australia—aim to design and build the world's first synthetic yeast genome (consisting of 16 chromosomes)**.

The laboratory benches of a dozen Sc2.0 research groups around the world (USA, UK, China, Singapore and Australia) are strewn with pieces of a complex 6,000‐piece (6,000‐gene) jigsaw puzzle comprising the genetic make‐up of *S. cerevisiae* 3. The pieces have been sorted into 16 piles (chromosomes) by colour, shape and size and divided between the puzzle masters (Fig 4). The challenge is to recreate the guide picture on the front of the box—a round‐to‐ovoid single‐celled fungus, 50–10 μm in diameter and compartmentalised like most other eukaryotic cells, including an encapsulated nucleus. While remaining true to this “blueprint”, each of the collaborating laboratories must meet the challenge of designing, building and interlocking the pieces of the *S. cerevisiae* genome puzzle in order to, for the first time, completely rebuilt a eukaryote's genome. Each piece of the puzzle is essential if a complete picture is to be produced, and the Sc2.0 team is working to have it in place by 2018.

As the Sc2.0 project is progressing, genome engineering technologies are being advanced at a rapid pace while important fundamental biological intricacies of yeast cells are being figured out.

**Figure 5:**
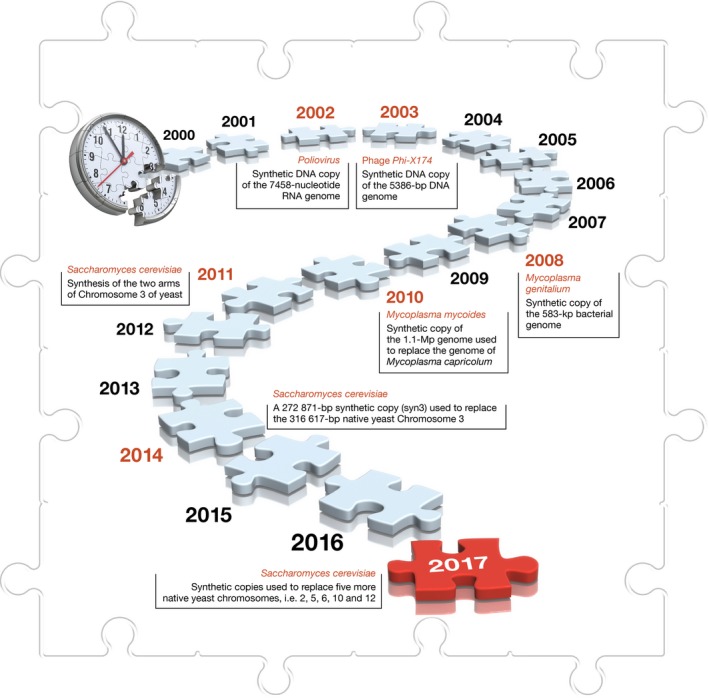
**Key milestones in terms of the synthesis of viral and bacterial genomes inspired the idea to chemically synthesise the 16 chromosomes of the yeast** ***Saccharomyces cerevisiae*** **and replace the native chromosomes with the synthetic chromosomes**.

**Figure 6:**
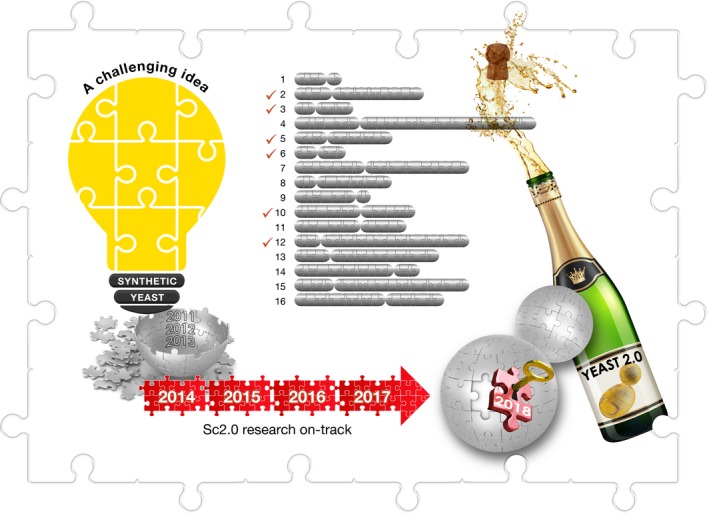
**The collaborative** ***Yeast 2.0*** **(** ***Sc2.0*** **) project, which commenced in 2011, has made significant progress with 6 of the 16 chromosomes synthesised—well on track to have all the native chromosomes of the yeast** ***Saccharomyces cerevisiae*** **replaced by 2018**.

The design of the Sc2.0 genome draws on data from the genome sequence first announced in 1996 for a haploid laboratory strain (S288c) of *S. cerevisiae*. The ~12 Mb (non‐redundant) to ~14 Mb (total) genome sequence carries approximately 6,000 genes of which about 5,000 are individually non‐essential. The 6,000 genes are distributed along 16 linear chromosomes of varying length (200–2,000 kb). The first step towards designing and building the *S. cerevisiae*'s genome was taken in 2011 with the synthesis of the two arms of Chromosome 3—the third smallest *S. cerevisiae* chromosome 2, 3. This opened the way for the synthesis of Chromosome 3 in full in 2014. Earlier this year, the synthetic versions of five more *S. cerevisiae* chromosomes have been published (Fig 5) 3. It is expected that all 16 chromosomes will be synthesised by the end of this year. The Sc2.0 project is thus on track to consolidate the 16 chemically synthesised chromosomes into a single cell of *S. cerevisiae* by 2018 (Fig 6).

This consolidated Sc2.0 genome was designed from the start to be fully customisable so that researchers will be able to ask otherwise intractable questions about the fundamentals of chromosome structure, organisation, function and evolution, as well as gene content, function of RNA splicing and the role of small RNAs in yeast biology 3. The guiding design principles for the Sc2.0 genome aspired to balance a desire to preserve the phenotype of the wild‐type yeast strain while incorporating inducible flexibility for further manipulation and minimising instability resulting from the repetitive nature of yeast's native genomic DNA. These principles for the design, construction, analysis, fitness testing and curation are most likely to be scalable to future synthetic work on the larger genomes of plants, animals and humans 3.

Wine yeast researchers expect to gain much knowledge by borrowing some of the Sc2.0 puzzle pieces to build their favourite wine yeast strains.

The final Sc2.0 genome is therefore designed, curated, streamlined and reorganised to encode a slightly modified genetic code 3. To facilitate the assembly of the synthetic chromosomes, specific base substitutions within some of the open reading frames (ORFs) are included to introduce necessary or remove inconvenient enzyme recognition sites. In addition, recognisable PCRTags—short recoded sequences within certain ORFs facilitating a polymerase chain reaction (PCR)‐based assay—are also included in the design of the Sc2.0 genome so that the synthetic DNA can be distinguished from native DNA 3. Other important modifications include the addition of many *lox*Psym sites for future genome scrambling; all TAG stop codons are recoded to TAA; all repetitive and dispensable sequences, such as *Ty* transposons, introns, subtelomeric regions and silent mating‐type loci (*HML* and *HMR* located on Chromosome 3) are omitted from the design; and all tRNA genes are relocated to a novel neochromosome 3. The expectation is that these designer changes would not cause any fitness defects but would allow a whole gamut of future genome manipulations and research opportunities. To date, about 75% of the DNA synthesis is complete and built into discrete strains by the various Sc2.0 teams.

History was made with the recent successful development of the world's first semi‐synthetic wine yeast as a “demonstrator” project.

As the Sc2.0 project is progressing, genome engineering technologies are being advanced at a rapid pace while important fundamental biological intricacies of yeast cells are being figured out. By the end of this project, it would be known, for example, whether removing all introns and transposable elements will affect cell fitness and whether the relocation of all tRNA genes to a 17^th^ mini‐neochromosome will disadvantage the genetic processes and protein synthesis machinery of the redesigned haploid S288c laboratory strain of *S. cerevisiae*. These are just a few examples of the puzzling questions that are being answered as the Sc2.0 picture emerges.

## Customising wine yeast under challenging conditions

From a wine scientist's viewpoint, another perplexing question is whether some of fundamental insights from the Sc2.0 yeast strain can be extrapolated to robust industrial wine yeast strains 2, 5, 6. Wine yeast researchers expect to gain much knowledge by borrowing some of the Sc2.0 puzzle pieces to build their favourite wine yeast strains. Since yeast fermentation is a centre piece in the process of winemaking, there is much to be gained by unlocking the genetic secrets that make different wine yeast strains perform differently. By understanding the fundamentals, the possibilities could be expanded by redesigning some wine yeast strains’ natural jigsaw puzzle pieces or inventing totally new ones. The objective of such strain development programmes would be to provide winemakers with a diverse array of wine yeast strains. Each strain would be specifically tailored to produce particular wine styles identified for various markets and market segments the world over.

Not all yeast strains are equally able to catalyse rapid, complete and efficient conversion of grape sugars to ethanol, carbon dioxide and other minor, but important metabolites—acids, alcohols, carbonyls, esters, terpenes, thiols and so on without the development of off‐flavours (e.g. hydrogen sulphide, volatile acids and volatile phenols) 2, 5, 6. Wine yeasts can differ widely in terms of their robustness, fermentation efficiencies and sensory properties, and performance depends on the specific composition of a particular grape juice and specific fermentation conditions and techniques used by the winemaker. During the past three decades or so, a wide variety of strain improvement techniques have been harnessed to optimise fermentation performance, robustness, spoilage control, processing efficiency, product wholesomeness and sensory quality 2, 5, 6.

Non‐genetic modification techniques include *hybridisation* (mating or cross‐breeding), *mutagenesis* (induction of mutations by exposure to mutagenic chemicals or ultraviolet radiation) and *adaptive evolution* (crossing and back‐crossing of selected mutants) 2. Several hybrid and mutant strains have been used successfully in global commercial winemaking. Consumers had no hesitation embracing the many award‐winning wines produced with rapid‐fermenting and aroma‐enhancing hybrid strains originating from mating and cross‐breeding, or the many fault‐free wines produced with mutants that no longer produce off‐flavours, such as hydrogen sulphide, volatile acidity and volatile phenols 2, 5, 6.

**Figure 7:**
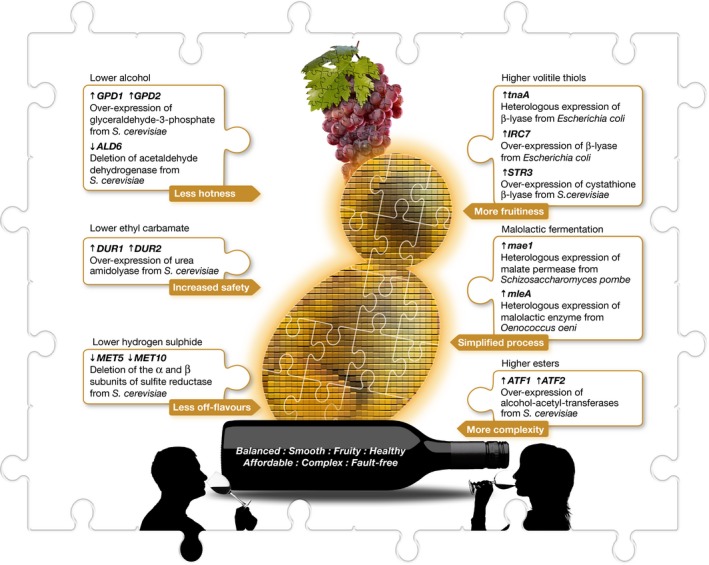
**Examples of wine yeasts bioengineered for improved robustness, fermentation efficiency and sensory attributes**.

Such broad‐based acceptance by producers and consumers is, however, not the case for strains generated by genetic engineering. More than 10 years ago, the first two GM wine yeast strains, ML01 and ECMo01, which met all regulatory requirements, were commercialised in the USA, Canada and Moldova 7, 8. Despite the proven success in winemaking trials and the clear benefits to both producers and consumers of the ML01 malolactic strain and the ECMo01 low‐ethylcarbamate strain, there is yet to be widespread uptake of these wine yeasts in commercial winemaking. ML01 and ECMo01 are not the only GM wine yeasts twiddling their budding thumbs at the entrances of wineries and cellar doors (Fig 7). Several robust and flavour‐active strains have been developed to mitigate stuck fermentations during problematic hot vintages and to create market‐driven wines with desired alcohol levels 6, 9 and aroma profiles 2, 5, 6. So far, the well‐orchestrated anti‐GMO campaigns, the furore over the labelling of GM food products and associated market sensitivities have deterred winemakers to take full advantage of science and the opportunities afforded by genetic engineering, and now more recently, by genome engineering.

By solving fundamental yeast jigsaw puzzles over a glass of wine, we might well acquire the ability to design the ultimate wine yeast genome model…

While the wine industry is caught up in the scientific and cultural maelstrom of the “wonders and terrors” of GMOs and SSOs, researchers continue to mine DNA sequencing data for the responsible design, synthesis and/or editing of wine yeast genomes with huge potential benefits for producers and consumers alike. On one hand, anti‐GMO/SSO campaigners and uninformed traditionalists might dismiss such research as an “unwanted reality” that will eventually vanish into thin air. However, future‐focussed innovators, on the other hand, are highly supportive of these research efforts because they recognise that it generates invaluable insights into the molecular intricacies of wine yeast cells. Akin to what research into the Higgs boson elementary particle is revealing about the “Standard Model” of particle physics, synthetic genomic experimentation is illuminating the biomolecular mysteries of wine yeast cells. Factually correct information and knowledge gained from such fundamental research and evidence‐based data are the only way to counteract ideologically driven doomsday prophecies, exaggerated fantasies, empty promises and guesswork about the future of SSOs.

## Uncorking a raspberry‐tasting Chardonnay produced by a semi‐synthetic wine yeast

**Figure 8:**
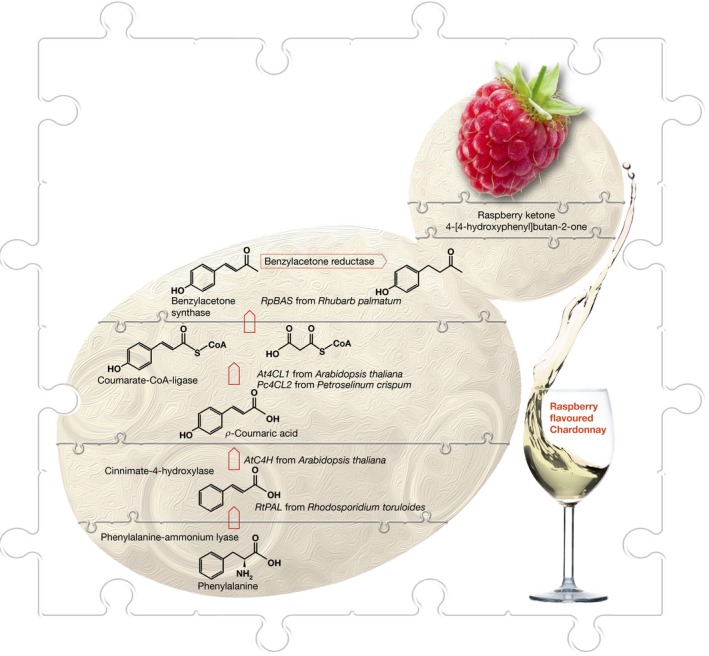
**The first semi‐synthetic wine yeast capable of producing Chardonnay wine with a raspberry aroma**.

History was made with the recent successful development of the world's first semi‐synthetic wine yeast as a “demonstrator” project. A cassette of four synthetic genes encoding the production of a highly desirable fragrant raspberry ketone—4‐[4‐hydroxyphenyl]‐butan‐2‐one—was embedded into the genetic blueprint of a wine yeast strain (Fig 8) 10. This phenylpropanoid is the primary aroma compound found in several fruits, vegetables and berries, including raspberries, blackberries, grapes and rhubarb, but, owing to the low concentrations present in these plants, it is not economical to extract this flavoursome compound from its natural sources. However, thanks to market preferences, chemically manufactured derivatives of this flavouring agent fetch much lower prices than the naturally derived form. This led to early attempts to produce raspberry ketone from *p*‐coumaric acid in heterologous bacterial and yeast strains. Yet, the high cost of *p*‐coumaric acid as a substrate and the trace amounts of raspberry ketone obtained in these GM strains prevented commercial production of this phenylpropanoid as a food‐grade flavouring agent. The missing puzzle pieces in this work are the ability to eliminate the requirement for supplementing the culture medium with expensive *p*‐coumaric acid and to increase the yield of 4‐[4‐hydroxyphenyl]‐butan‐2‐one 10.

Solving such a scientific puzzle starts with the unravelling of the phenylpropanoid biosynthetic pathway. This pathway commences with the conversion of phenylalanine to *p*‐coumaric acid via cinnamate or directly from tyrosine to *p*‐coumaric acid. Conversion of *p*‐coumaric acid to raspberry ketone requires three additional enzymatic steps including a condensation reaction between coumaroyl‐CoA and malonyl‐CoA. To design a biosynthetic pathway for the *de novo* production of 4‐[4‐hydroxyphenyl]‐butan‐2‐one in a wine yeast, the following codon‐optimised genes were chemically synthesised and integrated into the *HO* locus of a wine yeast strain (AWRI1631): the phenylalanine ammonia lyase from an oleaginous yeast, *Rhodosporidium toruloides*; the cinnamate‐4‐hydroxylase from *Arabidopsis thaliana*; and the coumarate CoA ligase 2 gene from parsley, *Petroselinum crispum*, fused by a rigid linker to the benzalacetone synthase from rhubarb, *Rheum palmatum*. This semi‐synthetic organism was equipped to produce raspberry ketone at concentrations almost two orders of magnitude above its predicted sensory threshold in Chardonnay grape juice under standard wine fermentation conditions, while retaining the ability to ferment the grape must to dryness 10.

The primary goal of this research project was not to produce raspberry‐tasting Chardonnay at a commercial scale. The objective was to hone our synthetic biology skills and to expand our toolkit with which we can advance fundamental understanding and provide solutions to the many riddling questions of flavour‐active wine yeast puzzles. By solving fundamental yeast jigsaw puzzles over a glass of wine, we might well acquire the ability to design the ultimate wine yeast genome model, thereby paving the way for further improvement of wine quality and consumer acceptance while minimising resource inputs, production costs and environmental impact.

## Conflict of interest

The author declares that he has no conflict of interest.
